# An unusual clinical manifestation of coronavirus disease 2019 in a woman with twin pregnancy: a case report

**DOI:** 10.1186/s13256-022-03377-9

**Published:** 2022-04-19

**Authors:** Laleh Eslamian, Seyedeh Noushin Ghalandarpoor-Attar, Azadeh Shabani, Seyedeh Mojgan Ghalandarpoor-Attar

**Affiliations:** 1 Obstetrics and Gynecology Department, Shariati Hospital, Tehran University of Medical Sciences, Tehran, Iran; 2 Obstetrics and Gynecology Department, Baqyiatallah Hospital, Baqyiatallah University of Medical Sciences, Tehran, Iran; 3 Obstetrics and Gynecology Department, Taleghani Hospital, Shahid Beheshti University of Medical Sciences, Tehran, Iran; 4 Obstetrics and Gynecology Department, Baharloo Hospital, Tehran University of Medical Sciences, Tehran, Iran

**Keywords:** Pregnancy, Cerebral vein thrombosis, COVID-19

## Abstract

**Background:**

Although coronavirus disease 2019 affects mainly the respiratory system, as time passes and our understanding of the disease improves, many nonrespiratory clinical manifestations such as thromboembolic events have been shown to occur with or without respiratory tract involvement.

**Case presentation:**

We present the case of a 21-year-old gravid 3, live 1, abortion 1 Iranian woman pregnant with twins in her early first trimester. Her initial chief complaint was headache that gradually increased in intensity. Eventually, cerebral vein thrombosis was confirmed. Although the patient first manifested with neurological involvement, she developed upper respiratory symptoms soon after, and then nasopharyngeal polymerase chain reaction test returned positive.

**Conclusion:**

Any neurological complaints in pregnant women during the current coronavirus disease 2019 pandemic should raise suspicion for the presence of significant cerebral thrombotic or ischemic events, even if the patient has no complaint of respiratory tract involvement and/or when an initial nasopharyngeal polymerase chain reaction test is negative.

## Introduction

Cerebrovascular events are acute complications of coronavirus disease 2019 (COVID-19), and cerebral stroke occurs in 2–6% of hospitalized patients [1]. Cerebral vein thrombosis (CVT) is a rare condition with an incidence of 0.2–1.75 per 100,000 people each year, according to data from Europe, Australia, Iran, and Hong Kong [2, 3]. Its known risk factors include female sex, infection, hypercoagulability, and pregnancy [4]. COVID-19 predisposes patients to thromboembolic events by inducing inflammation, platelet activation, endothelial damage, and stasis [5]. Furthermore, in Europe there have been some cases of CVT after COVID-19 vaccination, termed vaccine-induced thrombocytopenic thrombosis [6, 7]. It is not known whether COVID-19 activates a coagulation cascade or directly causes CVT [8]. Here we report a case of COVID-related CVT in a 21-year-old woman pregnant with twins.

## Case presentation

A 21-year-old Iranian woman with dichorionic–diamniotic spontaneous twin pregnancy presented to our obstetric emergency department complaining of severe headache on 6 April 2021. She was in the eighth week of gestation and had a history of previous uncomplicated term pregnancy and an early first-trimester abortion. Her body mass index (BMI) was 21.4 kg/m^2^, and she was suffering from unilateral right-sided pulsatile and throbbing occipital headache radiating to her ipsilateral half of the face since a week before her referral. Her headache intensity had increased since its onset, and simple analgesics had not been effective. She had no history of prepregnancy headache, and her only complaint during pregnancy was mild-to-moderate nausea and infrequent vomiting.

The patient had visited her obstetrician 2 days before presenting to the emergency department. The obstetrician had requested a nasopharyngeal COVID-19 polymerase chain reaction (PCR) test with suspicion of COVID-19 infection due to the COVID-19 pandemic condition, despite lack of its other common signs and symptoms. However the result was negative. Upon her arrival, she was afebrile, in stable hemodynamic condition, and completely conscious. Neurological examination did not reveal any abnormal finding. Because of suspected cerebrovascular events, the patient underwent magnetic resonance imaging (MRI) and magnetic resonance venography (MRV) of the brain. On the brain MRI, a large parenchymal hematoma was observed in the right temporal lobe with peripheral white matter edema and 2 mm of midline shift. On the MRV, right transverse and sigmoid dural sinuses were engorged on the T2-weighted images and showed diffuse filling defects compatible with cerebral vein thrombosis (CVT).

**Fig. 1:**
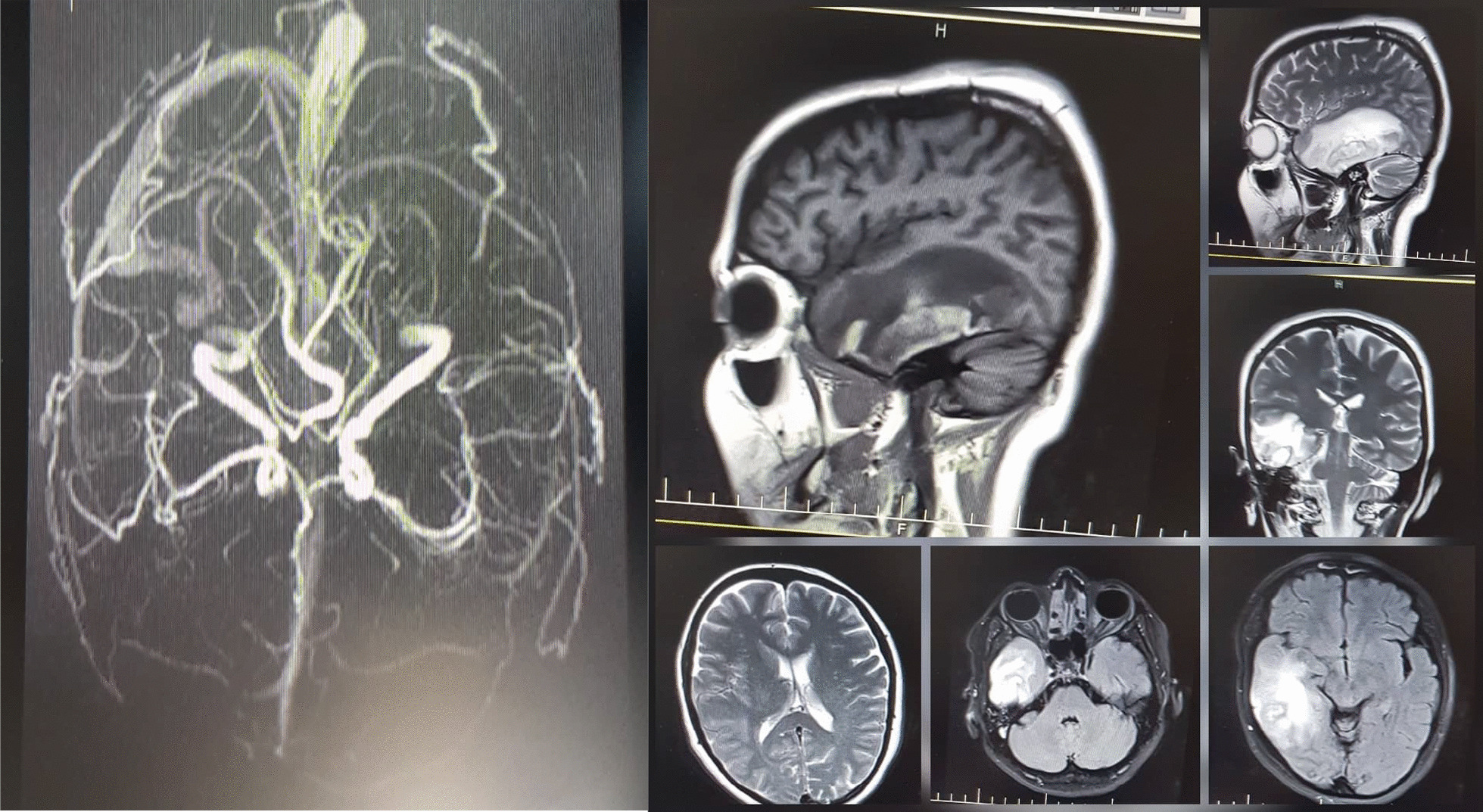
Patient’s brain MRI–MRV. The brain MRI (right) shows a large parenchymal hematoma in the right temporal lobe with peripheral white matter edema with 2 mm of midline shift. The MRV (left) shows engorgement of the right transverse and sigmoid dural sinuses on T2-weighted images and diffuse filling defects compatible with cerebral vein thrombosis

There was thrombosis involving the right inferior anastomotic vein (Labbé vein), too. Small patchy areas of filling defects in the superior sagittal and left transverse and sigmoid sinuses were also seen in favor of partial thrombosis. Fortunately, deep cerebral veins seemed to be intact. However, the straight sinus was partially obliterated (Fig. 1). Hence, full anticoagulation began with enoxaparin 1 mg/kg, and levetiracetam 500 mg twice a day was added for seizure prophylaxis. In addition, acetaminophen 500 mg every 6 hours was administered for pain control. The next day, her headache severity was obviously decreased; no new-onset focal neurological deficit was found, but she experienced a sore throat and flu-like symptoms. Since she had been in touch with a COVID-19-infected person recently, we repeated the nasopharyngeal PCR test despite her first negative test result (Table 1).

**Table 1:** Laboratory tests

Test	Result	Test	Result
WBC	7600/μL	Fibrinogen	287 mg/dL (NL)
RBC	4.39 × 10^6^/μL	D-dimer	0.8 μg/mL (NL)
Hb	12.6 g/dL	FDP	4 μg/mL (NL)
MCV	80 fL	BUN	9 mg/dL
Plt	147,000/μL	Cr	0.7 mg/dL
PTT	34 s	Na	141 mmol/L
PT	12.1 s	K	4.1 mmol/L
INR	1.02	AST	31 U/L
TSH	0.8 mIU/L	ALT	34 U/L

WBC: white blood cell count, RBC: Red blood cell count, Hb: Hemoglobin, MCV: Mean corpuscular volume, Plt: Platelet, PTT: Partial thromboplastin time, PT: Prothrombin time, INR: International normalized ratio, TSH: Thyroid stimulating hormone, FDP: Fibrinogen degradation products, BUN: Blood urea nitrogen, Cr: Creatinine, Na: Sodium, K: potassium, AST: Aspartate aminotransferase, ALT: Alanine aminotransferase

Surprisingly, her PCR test result returned positive. Hence, we began remdesivir 200 mg intravenously, then 100 mg daily plus dexamethasone 8 mg intravenously twice daily. To confirm COVID-associated CVT, all the inherited and acquired thrombophilia (prothrombin gene mutations, methyl-tetrahydrofolate reductase mutations, anti-thrombin III deficiency, factor V Leiden, protein S and C level, and antiphospholipid antibodies) were checked, and no abnormalities were found. Our patient showed a gradual decrease in her white blood cell (WBC) count and platelet (Plt) count on serial complete blood counts (CBCs) during her first 4 days of admission, while her peripheral blood smear did not show any abnormalities (Table 2). All the viral hepatitis tests (hepatitis A, B, and C) were negative, and no organomegaly or lymphadenopathy was evident on ultrasound imaging. Her blood group was O-negative, and direct and indirect Coombs test as well as Coombs–Wright test were also negative. Thus, the most probable reason for bicytopenia was COVID-19 infection or drug-induced adverse effects. Subsequently, we discontinued levetiracetam. Since then, WBC and Plt count began to rise. Our patient never complained of dyspnea, and her peripheral blood pulse oximetry always showed O_2_ saturation above 98%. The patient was discharged to home 6 days after her hospital stay without any complications; both fetuses had normal fetal cardiac activity, and no retroplacental hematoma was evident on ultrasonography. The patient had biweekly prenatal and monthly neurological visits during the first and second trimesters, followed by weekly visits in the third trimester. She underwent an uncomplicated cesarean section due to a cephalic-breech presentation twin pregnancy at 38 weeks of gestation, and both healthy newborns and the mother were discharged 72 hours postpartum. Full anticoagulation was continued according to neurological consultation for 6 weeks, then all thrombophilia tests were rechecked; as no abnormality was found, anticoagulation was discontinued.

**Table 2:** Serial complete blood cell count (CBC)

CBC	Day 1	Day 2	Day 3	Day 4	Day 5	Day 6	Day 7
WBC (/μL)	7600	5670	3180	2300	2900	3390	4200
Hb (g/dL)	12.6	11.5	11.2	11.2	11.7	11.6	12
Plt (/μL)	150,000	127,000	117,000	108,000	124,000	149,000	149,000

WBC: white blood cell count, Hb: Hemoglobin, Plt: Platelet count

## Discussion

It has been confirmed that COVID-19 affects mainly the respiratory system, but many other organs, including those of the nervous system, can be involved [9]. Thrombotic events are other clinical features of COVID-19 infection, but their exact mechanism has not been well understood [5, 10, 11]. On the other hand, pregnancy and postpartum period predispose the patient to thromboembolic events. CVT often occurs in the third trimester or, more commonly, postpartum. Surprisingly, CVT occurred in our patient in the early first trimester. Although she had a twin pregnancy, among all other risk factors, she had only experienced mild-to-moderate nausea and infrequent vomiting episodes that had not led to hospital stay.

There was no history of thromboembolic events, stillbirth, or recurrent abortions in her first- or second-degree relatives, and all inherited and acquired thrombophilia assessments were negative, too. It seems that COVID-19 infection manifested with neurological symptoms initially while her PCR test was negative. However, after upper respiratory tract involvement and flu-like syndrome occurrence later on her second day of admission (9 days after neurological manifestation onset), the PCR test result returned positive. Additionally, it is worth mentioning that, as the patient had no complaints of lower respiratory tract involvement and her COVID-19 infection had been confirmed via nasopharyngeal PCR, no spiral chest computed tomography scan was performed, although minor or subclinical COVID-19 pulmonary involvement could not be ruled out definitively. However, as Doppler ultrasonography of the lower extremities, electrocardiogram, and echocardiography were all unremarkable, no further imaging was done to investigate pulmonary thromboembolic complications.

Gunduz *et al.* also reported the case of a 35-week pregnant woman diagnosed with CVT and a positive PCR test result. She had no fever or respiratory symptoms and high level of fibrinogen and D-dimer (compared with our patient). Her MRI–MRV images revealed partial venous thrombosis in left transverse sinus, and her Plt count decreased from 107,000/μL to 67,000/μL (same as in our patient). Postpartum chest computed tomography scan revealed pulmonary involvement [12].

In a study on 184 COVID-19-positive patients in intensive care unit, 31% had thrombotic events, highlighting the importance of thromboprophylaxis [13]. Cerebrovascular events are acute complications of COVID, and cerebral stroke has been reported in 2–6% of hospitalized COVID-infected patients. In a retrospective study on 219 COVID-19 infected patients, Li *et al.* reported that 4.6% of the patients had cerebral ischemia symptoms and 0.5% had cerebrovascular hemorrhagic symptoms. These neurologic symptoms had occurred 10 days after the infection on average. These patients’ mean age was higher (75.7 ± 10.8 years versus 52.1 ± 15.3 years), and they had more cardiovascular risk factors [14]. However, in this current case, COVID-19 infection first manifested with neurological symptoms, and subsequently, upper respiratory symptoms appeared.

In their case series of COVID-19-related CVTs, Ostovan *et al.* found that four out of six patients had no respiratory symptoms and five out of six patients had clinical manifestations of CVT and COVID-19 infection concomitantly [15]. In this presented case, the first nasopharyngeal PCR was negative, which might have been due to false negativity. Thus, it seems that there might be a relation between respiratory signs and risk of CVT, as observed in our case.

Understanding the mechanism of COVID-related thromboembolic events requires more investigations [8]. The patients’ mean age is higher in COVID-related CVTs compared with non-COVID-related CVTs [16]. However, our patient was only 21 years old.

Schulz *et al.* also reported 62 patients with cerebral events in close temporal proximity to their COVID-19 vaccination. Of 45 CVT cases, 35 were women (77.8%), 36 patients were younger than 60 years old, intracranial hemorrhage was observed in four cases (6.4%), and nine cases (14.3%) had primary cerebral ischemia. The median time interval from the last administered vaccine shot to the first neurological complications was 9 days. They estimated an incidence rate of 6.5–8.8 cerebrovascular events per 100,000 people annually within 1 month from administering the first dosage. They also reported four patients with primary intracerebral bleeding without imaging evidence of CVT [17]. Our case had intraparenchymal hematoma and CVT concomitantly.

CVT during pregnancy most often involves the superior sagittal sinus, but it may occur anywhere in cerebral venous circulation [18, 19]. We documented complete right transverse and sigmoid sinuses, Labbé vein thrombosis, partial superior sagittal, left transverse and sigmoid sinus thrombosis, and intraparenchymal hematoma in our patient.

We show a case of twin pregnancy combined with systemic inflammation and hypercoagulability associated with COVID-19 that resulted in cerebral vein thrombosis, which was followed by upper respiratory tract involvement later on.

## Conclusion

Headache is clinical presentation of COVID-19 infection that is easily neglected. However, as infection can predispose patients to cerebrovascular events such as CVT, it seems reasonable that during the COVID-19 pandemic any neurological complaints, even a simple headache in a young, low-risk pregnant woman, should raise clinical suspicion of life-threatening conditions such as cerebrovascular events. Proper diagnostic approach should be considered to prevent related mortality and morbidities.

## Data Availability

All data generated or analyzed during this study are included in this published article (and its additional information files)

## Abbreviations

CVT: Cerebral vein thrombosis
BMI: Body mass index
PCR: Polymerase change reaction
MRI: Magnetic resonance imaging
MRV: Magnetic resonance venography
CBC: Complete blood cell count
WBC: White blood cell
Hb: Hemoglobin
Plt: Platelet
